# Unreliable evidence to support a vector regulation hypothesis for *Xylella fastidiosa*–leafhopper interactions

**DOI:** 10.1128/aem.00854-25

**Published:** 2025-07-31

**Authors:** Rodrigo P. P. Almeida

**Affiliations:** 1 Department of Environmental Science, Policy and Management, University of California, Berkeley 1438, Berkeley, California, USA; The University of Tennessee Knoxville, Knoxville, Tennessee, USA

**Keywords:** biofilm development, *Xylella fastidiosa*, insect-borne

## LETTER

*Xylella fastidiosa* is an important bacterial plant pathogen transmitted by xylem sap-feeding insects. In the Americas, sharpshooter leafhoppers (Hemiptera, Cicadellidae) are the most ecologically relevant group of vectors. As summarized by Backus and Shugart (1), *X. fastidiosa* persistently colonizes the cuticular surface of the foregut of vector species. This phenomenon was first reported by Severin in 1949 (2), when he showed that insects remained infected over 100 days post-exposure to infected plants. Here, Backus and Shugart used scanning electron microscopy (SEM) and quantitative PCR (qPCR) to study patterns of *X. fastidiosa* colonization of vectors over 7 days of insect exposure to infected host plants. The experimental design used makes it difficult to determine how the data support the conclusions. To study the temporal dynamics of biofilm formation and dislodgment from the foregut of vectors, the authors confined insects on *X. fastidiosa*-infected plants for pathogen acquisition for up to 7 days. Each day for 7 days of continuous exposure, when qPCR was used, or at 4 and 7 days of continuous exposure for SEM, insects were removed from infected plants and processed for *X. fastidiosa* quantification or microscopy observations. Afterward, the results were contextualized as phases of biofilm development in the foregut of insects over time. And here lies the crux of the problem: by continuously exposing insects to infected plants over 7 days, it is impossible to determine (i) when pathogen acquisition occurred and (ii) how many times pathogen acquisition occurred. Even if only one acquisition (vector colonization) event occurred during the exposure period, it is not possible to know when it happened; for example, for insects collected on the last day of exposure, did acquisition happen on the day before sample collection, or did it happen 6 days prior? In other words, one does not know when biofilm development initiated, or how many times it initiated over the evaluation period. An argument could be made for the continuous insect exposure to infected plants if that was common in nature, but under choice conditions, vectors of *X. fastidiosa*, particularly *Graphocephala atropunctata* on grapevines, avoid symptomatic plants as used in this work (3). One alternative experimental design to address the above-described concern, even if not perfect, would be to expose insects to infected plants for short periods of time, maybe up to 1 day of exposure for a 7-day biofilm development study, so that any acquisition event is limited to one specific time point. Afterward, maintaining the insects on new plants that are not infected with *X. fastidiosa* prior to testing would limit the likelihood of insects re-acquiring the pathogen, even if they inoculated these new plants; daily changing these intermediate plants would further reduce the likelihood of additional acquisition events occurring.
